# Joint injection practice variations in pediatric rheumatology – a global survey and call for action

**DOI:** 10.1186/s12969-020-00475-0

**Published:** 2020-10-17

**Authors:** Anita Dhanrajani, Raju P. Khubchandani

**Affiliations:** 1 Hospital for Sick Children, 8253, Burton Wing, 555 University Avenue, Toronto, ON M5G 1X8 Canada; 2 Pediatric Rheumatology, SRCC Children’s Hospital, 1-1A, Keshavrao Khadye Marg, Haji Ali, Haji Ali Government Colony, Mahalakshmi, Mumbai, Maharashtra 400034 India

**Keywords:** Arthritis, Juvenile, Injections, Intra-articular, Surveys and questionnaires

## Abstract

**Background:**

Intraarticular injections (IAI) were first reported in adult rheumatology in the 1950s and subsequently gained acceptance as a safe and efficacious treatment in Juvenile idiopathic arthritis (JIA). IAIs are now widely performed and recommended as the initial or only treatment of oligoarticular JIA and ancillary treatment of actively inflamed joints in other varieties of JIA. However, the performance of the procedure is currently not guided by standardized recommendations, and several practice variations are observed.

**Methods:**

This worldwide survey of pediatric rheumatologists (with 48.5% response from Pediatric Rheumatology International Trials Organization [PRINTO and Pediatric Rheumatology Collaborative Study Group [PRCSG] members) captures the differences in pre-procedural, procedural and post-procedural protocols and practices observed across the globe and asks the necessity of developing consensus in this area of Pediatric Rheumatology.

**Results:**

This worldwide survey of Pediatric Rheumatologists had a response rate of just under 50% and the views of about 42% who routinely performed the procedure. It captured the differences in IAI protocols and practices observed across the globe. Significant variations in practice were noted in use of Local anesthesia, choice, and dose of therapeutic agent for the intraarticular injection and use of ultrasound to guide injections. While some practice variations may be explained by institutional protocols in different parts of the world, the clinical implications of these are largely unknown and beg the need for further studies.

**Conclusions:**

Given these practice variations, the authors recommend further studies to explore the cost and clinical implications and subsequently work towards developing consensus plans to ensure uniformity in this widely used procedure in Pediatric Rheumatology.

## Background

Intraarticular injections (IAIs) are a common practice in Pediatric Rheumatology, specifically for the treatment of Juvenile idiopathic arthritis (JIA). The earliest published study on IAI use in pediatrics comes from Petty et al. in 1986 [1], although there are anecdotal reports of pediatric joint injections predating this [2]. Currently, IAI is an accepted initial primary or supplemental tool in management of chronic arthritis in children. The use of glucocorticoid IAI in newly diagnosed oligoarticular JIA is recommended as the initial therapy in the American College of Rheumatology (ACR) Recommendations for the Treatment of JIA: 2011, irrespective of disease activity and prognostic factors [3].

Several studies, including a systematic review and meta-analysis have clearly established the superior efficacy of Triamcinolone hexacetonide (TH) over Triamcinolone acetonide (TA) in equivalent doses in pediatric IAIs [4–6]. Despite this, TH continues to be unavailable in several countries and innovative methods of procuring it have been reported [7].

Despite the wide use of this procedure in chronic arthritis in children, there are no standard recommendations or guidelines on IAI practices in Pediatric Rheumatology.

In the absence of recommendations to guide IAIs, it is likely that several variations exist in the practice worldwide. The aim of this study was to capture these variations with a web-based questionnaire distributed to members of two major scientific communities – Pediatric Rheumatology International Trials Organization [PRINTO] in the European continent, and Pediatric Rheumatology Collaborative Study Group [PRCSG] in North America, whose membership additionally spans across other continents.

The objectives of the survey were to collect information regarding IAI protocols and practices and to explore the relationship of potential variation with physician demographic features. The ultimate aim is to bring attention to the worldwide variations in practices of this extremely common procedure, to encourage further studies to establish the clinical implications of these variations and work towards developing a set of global recommendations for IAI.

## Methods

### Questionnaire design

A 22-item questionnaire comprising three main sections was designed. Section A pertained to questions regarding procedural variations with respect to: setting of joint injection, number of joints injected, use of ultrasound guidance, choice and dose of therapeutic agent, availability of TH, anesthesia preferences, complications, techniques for prevention of complications, and post procedure practices. Section B addressed variations in practice for patients less than 5 years of age, and Section C was focused on physician demographic characteristics. The questionnaire was pilot tested by 3 pediatric rheumatologists prior to dissemination. The survey was created and disseminated using the web-based platform Survey Monkey. Ethics approval was obtained from Ethics Committee of Jaslok Hospital and Research Center, Mumbai, India.

### Subject selection

The survey link was disseminated via email to: PRCSG (*n* = 169), and PRINTO/PRES members (*n* = 568). The survey was live for 8 weeks with one email reminder sent at the end of week 4.

### Statistical analysis

Descriptive data are presented as frequencies. Associations between two variables was calculated using chi-square test or Fisher’s exact test. Graphpad Prism version 6.01 was used for data analysis.

## Results

### Responses

The overall response rate was 48.5% (358/737). 310/358 respondents (87%) routinely performed IAI in their clinical practice. The remaining 48 responses were excluded from the analysis as they did not perform IAI routinely. Thus, of the 737 members, 310 responses were captured in this survey (42.1%).

### Demographics of respondents

**Fig. 1:**
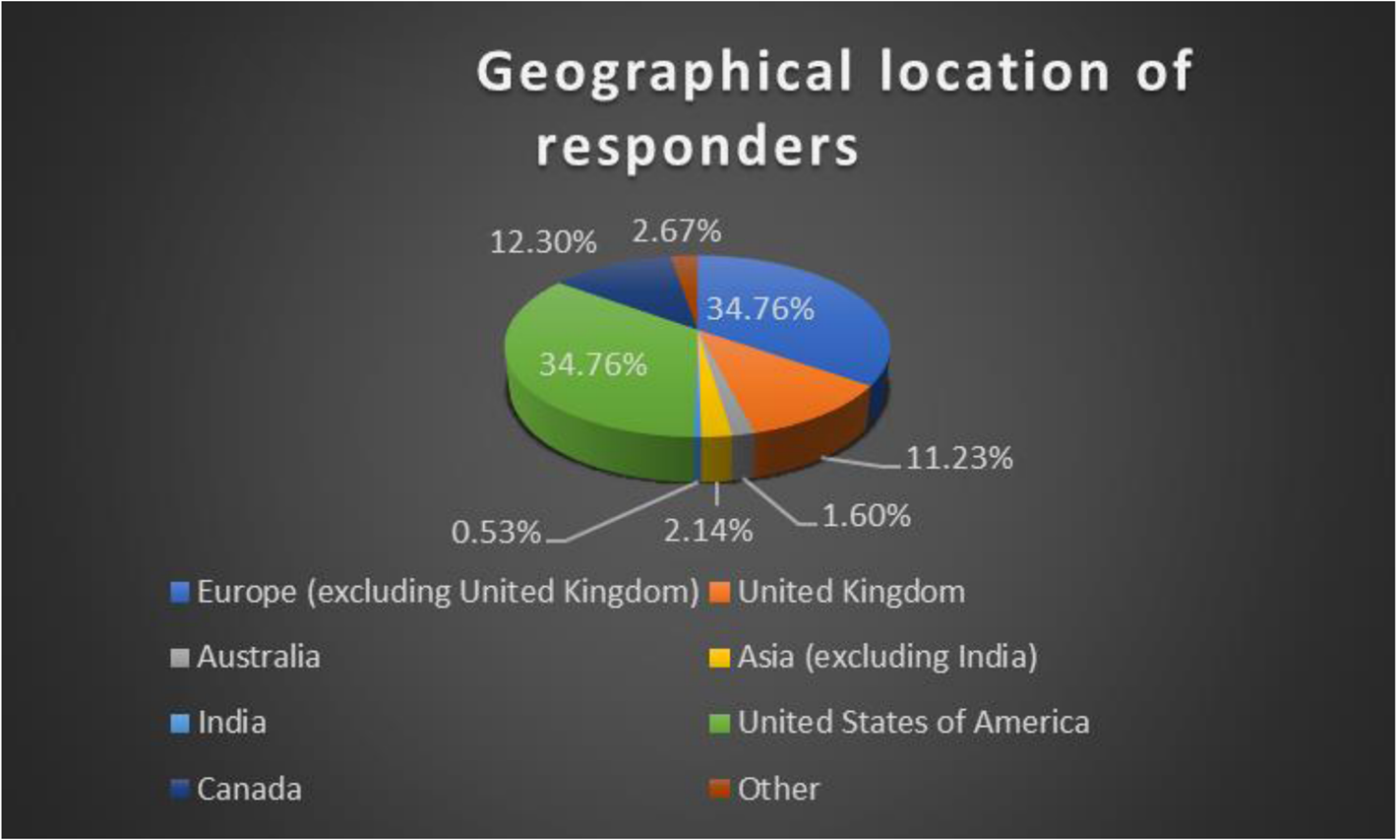
Geographical location of responders

The survey was distributed electronically to members of PRCSG and PRINTO groups with the aim to cover a wide geographical distribution. Table 1 provides baseline information about respondents and Fig. 1 shows the wide geographical distribution of the respondents. 195/327 respondents were formally trained in IAI. Other demographics such as age of the respondent, qualifications and credentials were not collected.

**Table 1:** Demographic characteristics of respondents

	Total respondents: 318 ^**a**^ N (%)	
**Number of years in Pediatric Rheumatology clinical practice**	**0–5 years**	**31 (9.8)**
	**5–10 years**	**68 (21.4)**
	**10–15 years**	**66 (20.8)**
	**> 15 years**	**153 (48.1)**

^a^Demographics were collected for all respondents

### Setting of IAI and use of anesthesia

Majority of the respondents indicated that the procedure was performed in an outpatient setting. 8% of respondents chose “other” which was further elaborated in comments as “setting differed based on the age of the patient and/or number of joints”.

Local anesthesia was the most popular choice amongst respondents (68%) and Eutectic Mixture of Local Anesthetics (EMLA cream) was the most used local anesthetic agent (39%). Use of sedation and anesthesia is depicted in Table 2. 22/87 respondents that indicated “other” mentioned the use of Nitrous oxide gas combined with oxygen for sedation in IAI.

**Table 2:** IAI setting and sedation

Use of sedation n (%)		Setting of IAI n (%)	
Oral sedation	52 (18.4)	Outpatient/Procedure room	159 (52.8)
Short anesthesia	137 (48.6)	Day care/Minor Operating room	97 (32.3)
Long anesthesia	6 (2.1)	Sonography suite	11 (3.7)
Local anesthesia		Major Operating room	10 (3.3)
Other	87 (30.9)	Other	24 (7.8)

### Number of joints injected and use of ultrasound

79.7% of respondents inject multiple joints in one sitting; the median number of joints injected per sitting was 4 (range 2–45). Data regarding use of ultrasound in general and for specific joints is presented in Table 3

**Table 3:** Ultrasound use for IAI

Use of ultrasound to guide IAI: n (%) Total ***n*** = 301		Frequency of ultrasound use in different joints Total ***n*** = 152	
Always	22 (7.3)	Hip	152 (100)
Selectively	135 (44.9)	Ankle	137 (90.1)
Never	144 (47.9)	Wrist	101 (66.5)
		Shoulder	63 (41.5)
		Small joints	44 (29)

### Therapeutic agent and dose

Ignoring availability, the medication of choice for IAIs was TH. However, more than 50% respondents reported that TH was either not available (41.9%) or sporadically available (9.51%) in their country. Table 4 depicts frequency of use of different therapeutic agents.

**Table 4:** Therapeutic agent of choice for IAI

Agent of choice	Respondents N (%)
Triamcinolone Hexacetonide (TH)	223 (78.5)
Triamcinolone acetonide (TA)	39 (13.7)
Methylprednisolone	10 (3.5)
Hydrocortisone	1 (0.4)
Others^a^	11 (3.9)

^a^Others: Betamethasone

**Fig. 2:**
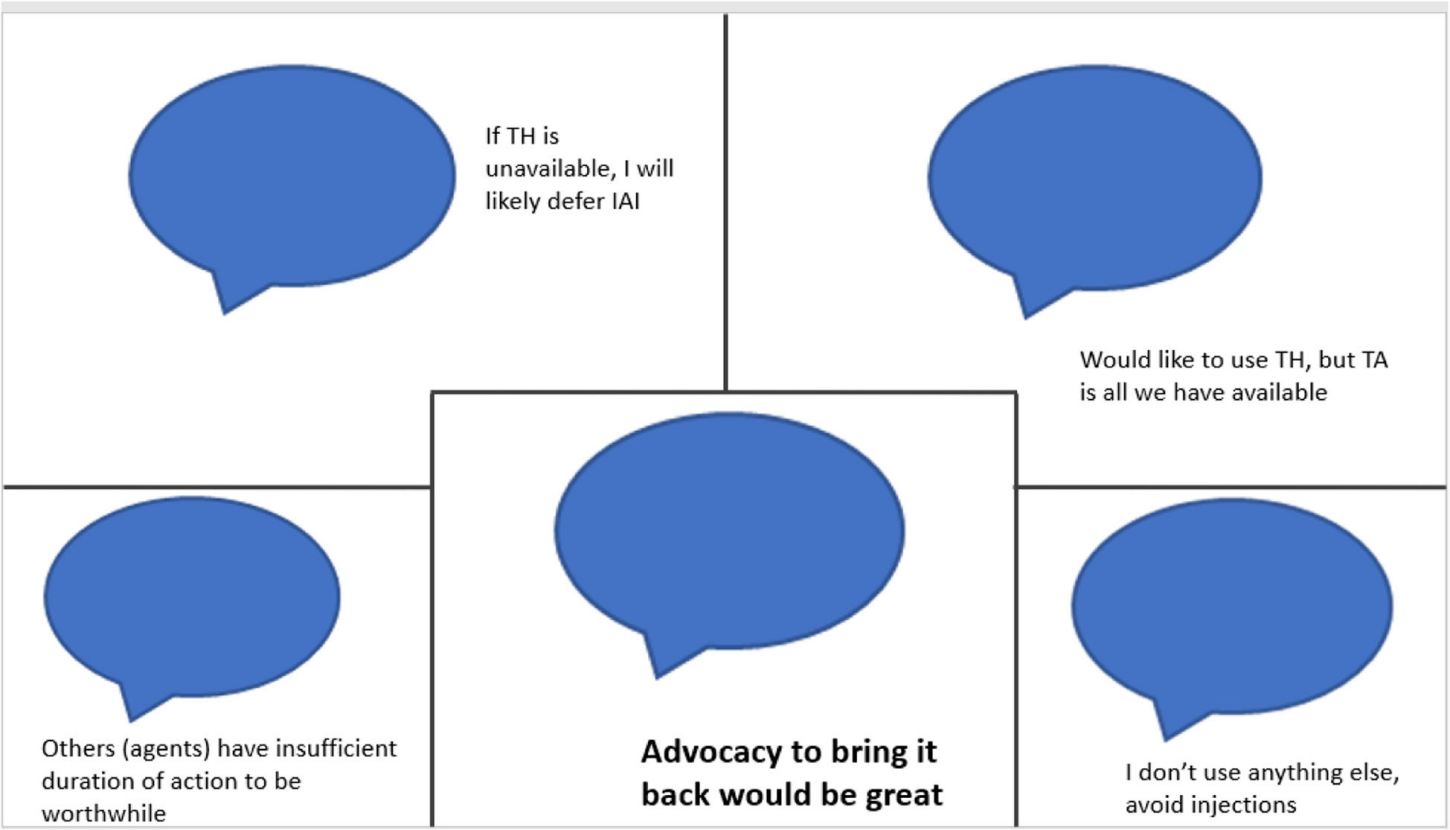
Comments regarding TH non-availability

Whereas most respondents indicated use of an alternative agent if TH was unavailable, several free text comments (Fig. 2) indicated that non-availability of TH is perceived as a significant barrier.

### Dose of steroid in TH equivalent

The most commonly used dose of steroid (TH equivalent) for large and small joint injections was 1 mg/kg and 0.5 mg/kg, respectively (*n* = 180 [64%] and *n* = 155 [55%], respectively) (Table 5).

**Table 5:** Dose of therapeutic agent

Dose in TH equivalent (mg/kg)	Response frequency: n (%)	
	Large joints	Small joints
0.5	24 (8.5)	155 (54.6)
1	180 (63.4)	36 (12.7)
1.5	12 (4.2)	0
2	29 (10.2)	1 (0.4)
Other ^a^	39 (13.7)	50 (17.6)

^a^Other: Absolute doses ranging from 10 to 30 mg

### Complications and monitoring

Complications reported by respondents are detailed in Table 6. The most reported complication was subcutaneous atrophy due to leaking of the therapeutic agent (79.1% of respondents). Techniques to prevent post-injection steroid leakage and subcutaneous atrophy included: reinjecting lidocaine (14.18%), quick withdrawal of needle (34.75%), combination of the above two (10.64%). 17% respondents reported no specific preventive measures. Amongst those who selected “other”, application of pressure, injecting normal saline, physiological serum, bupivacaine, air, limiting the volume injected, Z-track method and pressure application after needle withdrawal were reported.

**Table 6:** Complications of IAI

Reported complication	Total respondents: *N* = 282 N (%)
Subcutaneous atrophy (drug leakage)	223 (79.1)
Fat necrosis	56 (19.9)
Local bleeding	39 (13.8)
Anesthetic complications	14 (5)
Iatrogenic infections	7 (2.5)
Tendon rupture	3 (1.1)
Hypopigmentation (without atrophy)	4 (< 1)
Periarticular calcifications	3 (< 1)
Pain	6 (< 1)
Seizures	1 (< 1)
No complications	51 (18.1)

### Post-procedure monitoring and instructions

Most respondents indicated that they monitor the patient in the hospital until the effect of anesthesia wears off (77%). A minority of respondents indicated full day or overnight observation (6.4 and 4.6%, respectively).

### Age-related practices

47% respondents followed significantly different practices for children less than 5 years of age. Of these, the commonest age-dependent practice (72%) was choice of anesthesia (Table 7).

**Table 7:** Age dependent practice variations

Modified age-related practice for children < 5 years	Frequency: n (%) Total ***n*** = 131
Use of ultrasound guidance	31 (23.7)
Choice of anesthesia	94 (71.8)
Choice of setting for procedure (inpatient versus procedure room)	68 (51.9)
Number of joints injected	36 (27.5)
Duration of monitoring	22 (16.8)
Other (please specify)	3 (2.3)

### Associations

Variations in practices based on geographical location of the respondents and years of training was analysed with chi-square and Fishers exact tests.

A statistically significant relationship was observed between use of local anaesthesia (LA) and geographical location of USA versus UK. None of the other practices, such as use of ultrasound, the setting of IAI, number of joints injected in one setting, or age-related practice variations varied significantly by geographical location. There was no significant difference in practices based on years of clinical experience. Physicians who had received formal training in IAI appeared to follow different practices in the less than 5-year age group, as compared to physicians without formal training (Table 8).

**Table 8:** Results of Chi-square and Fisher exact test

	Frequency (%)		Chi-square value	***p***-value
**LA use**	USA: 49/65	UK: 10/21	5.681	**0.017**
	47	75		
**Variation in practices for age < 5 years**	Formal training in IAI	No formal training in IAI	-	**< 0.00001** (Fisher exact test)
	67	0		

*LA* Local Anesthesia

## Discussion

The American College of Rheumatology (ACR) guidelines for management of JIA [3] recommend joint injections with glucocorticoids as an important modality of treatment for active arthritis, regardless of concurrent therapy. For oligoarthritis (involvement of 4 or fewer joints), intraarticular injections are recommended as initial treatment or after failing 2 months of treatment with NSAIDs.

In a survey about treatment of Oligoarticular JIA in North America, 90% of physicians indicated use of IAI as either initial treatment or after failure of NSAIDs [8].

IAI in JIA is unequivocally recommended and widely used as the treatment of choice for active arthritis. However, to date, no recommendations regarding IAI practices have been published. In the recently published ACR guidelines of JIA, IAI receives a notable but small mention [9].

To the authors’ knowledge, no studies have attempted to explore the variable practices related to IAIs in pediatrics. While some factors such as the setting of joint injection and number of joints injected did not show much variability, several other practice variations in IAI were observed in our study.

Most importantly, the choice of therapeutic agent remains a widely varying decision, based mostly on lack of availability of TH. Small prospective trials and retrospective chart reviews have studied the efficacy of TA and TH in IAI and concluded that TH offers an advantage to TA, due to a longer duration of action [4, 5]. In a study by Eberhard et al. from New York, 794 IAIs were examined of which 422 were injected with TH and 372 with TA. TH proved more effective than TA with respect to the time to relapse for first injection (*p* < 0.001) [10]. ACR guidelines for management of JIA also strongly recommend TH over TA for IAIs based on reports from observational studies and clinical experience of the voting members of the committee. However, as demonstrated in the results of our survey, more than half of the respondents indicated difficulty in obtaining TH and either resorted to an alternative agent or deferred the procedure entirely. TH shortage remains an ongoing issue in several parts of the world [7] and has been reported in the FDA list of current drug shortages [11]. Advocacy initiatives based on the success story of some local advocacy groups [12] should be initiated and pursued to fill this gap in availability of TH.

Another major practice variation observed in this survey was use of ultrasound to guide joint injections. Almost half (47.9%) respondents indicated not using ultrasound either routinely or even selectively for IAI. There is data indicating effectiveness of radiologically guided IAIs particularly for complex joints [13, 14]. In our survey, we did not explore the reasons for under utilization of ultrasound. The use of ultrasound to assess joint remission in JIA is also documented in the literature [15].

IAI is a relatively safe procedure without major systemic side effects. The incidence of reported complications ranges from 2.6 to 8.3% [16, 17]. Some known minor complications of IAI include infections, skin atrophy, hypopigmentation, articular calcifications, and avascular necrosis [18].

Notably, 18% of our respondents had not witnessed a single complication following IAI.

The optimal time for post-procedure rest is controversial. In our study, only a minority of respondents observed the patient overnight or for 24 h. Although we did not specifically ask regarding resting or splinting an individual joint, the period of observation indirectly implies resting the patient (hence joint). A Cochrane review to determine the effects of rest following IAI in adults or children with arthritis concluded that there is some evidence for resting knees following IAI in adults, the findings must be extrapolated with caution in children with JIA [19].

### Limitations of the study

Surveys have an inherent limitation of differences in understanding and interpreting questions. Most questions in the survey were self-explanatory, however given the multilingual nature of the global respondents, there was an inherent risk of differences in understanding and interpreting questions. Although 42% (the number of respondents that use IAI routinely) would be considered a satisfactory response rate, there the rest that likely use IAIs in their practice but whose response could not be elicited as they could not be reached through this survey. It was beyond the scope of this study to explore the clinical and cost implications of these variations.

## Conclusions

IAI is a common modality of treatment in JIA but does not have established practice standards. Major variations were noted in the choice of therapeutic agent and use of ultrasound to guide IAIs in this study. Differing practices in a younger age group of patients was found to be significantly associated with the presence or absence of physicians’ formal training in IAI. Use of LA was significantly less frequent in some geographical areas, which may be explained by institutional or patient preferences, but needs to be further explored. The variations observed in this study beg for a worldwide collaboration to determine best practices and recommendations for this efficacious and safe procedure.

### Future directions

Further studies are needed to determine efficacious and cost-effective procedural practices for joint injections. The authors strongly recommend the development of a working group dedicated to developing a consensus statement on this extremely common, safe, and efficacious procedure in pediatric rheumatology. With COVID time teaching us the power of online collaboration this could be a relatively inexpensive exercise. Another important issue highlighted in this study, which may be within the realm of advocacy group agendas, is addressing the non-availability of TH in several parts of the world.

## Data Availability

The datasets used and/or analysed during the current study are available from the corresponding author on reasonable request.

## Abbreviations

IAI: Intra-articular Injection
JIA: Juvenile Idiopathic Arthritis
PRINTO: Pediatric Rheumatology International Trials Organization
PRCSG: Pediatric Rheumatology Collaborative Study Group
ACR: American College of Rheumatology
TH: Triamcinolone Hexacetonide
TA: Triamcinolone Acetonide
NSAIDs: Non-Steroidal Anti-inflammatory Drugs
CARRA: Childhood Arthritis & Rheumatology Research Alliance
LA: Local Anesthesia
